# Clinical characteristics, genotype-phenotype correlation, and prognostic follow-up of 48 Chinese children with hereditary spherocytosis

**DOI:** 10.3389/fmed.2026.1918209

**Published:** 2026-09-16

**Authors:** Fei Xie, Yu Gao, Lu Gan, Ping Hong, Shiyan Cao, Xiaoying Liu, Haihui Tong, Lei Lei, Bin Cai, Lin Zhou

**Affiliations:** Department of Pediatrics, The First Affiliated Hospital of Naval Medical University, Shanghai, China

**Keywords:** children, follow-up, gene mutation, hereditary spherocytosis, NGS

## Abstract

**Objective:**

This retrospective single-center observational study aimed to characterize clinical phenotypes and pathogenic mutation spectra in Chinese children with hereditary spherocytosis (HS), analyze genotype–phenotype correlations.

**Methods:**

Clinical and genetic data of 48 pediatric HS patients admitted to the First Affiliated Hospital of Naval Medical University from May 2016 to March 2024 were retrospectively analyzed. Clinical indices were compared across subgroups stratified by mutant genes and variant types. Forty patients were followed up via telephone to record post-discharge symptoms, therapies and complications such as cholelithiasis.

**Results:**

Forty-eight pathogenic variants were confirmed, including 13 novel mutations. SPTB (43.75%) and ANK1 (37.5%) predominated, and 28 patients had positive family histories. Moderate-severe anemia patients presented earlier diagnosis, more transfusions and lower MCHC. ANK1 variants correlated with higher reticulocyte levels and more transfusion dependence; splicing variants were linked to elevated reticulocytes and higher splenomegaly rates, while missense variants yielded milder manifestations. Among 40 followed patients, 12 received splenectomy with resolved hemolysis, no transfusions, improved exercise tolerance and unimpaired growth. No children had a height below P3, and growth profiles were similar between splenectomized and non-splenectomized patients.

**Conclusion:**

This is the largest Chinese single-center cohort with the longest reported follow-up of pediatric HS, featuring distinct local mutational profiles. This study enriches the Chinese HS mutation spectrum and guides clinical management, genetic counseling and future gene therapy research for pediatric HS.

## 1 Introduction

Hereditary spherocytosis (HS; MIM#612641) is an inherited disorder driven by congenital defects in erythrocyte membrane proteins, which ultimately trigger chronic hemolytic anemia as its core pathogenesis (1). Pathogenic variants mainly occur in five well-established causative genes: *ANK1*, *SPTB*, *SLC4A1*, *SPTA1* and *EPB42*. The majority of HS cases follow autosomal dominant inheritance, whereas a small proportion are inherited in an autosomal recessive pattern (2).

HS is typified by spherical red blood cells visible on peripheral blood smears and presents highly heterogeneous clinical phenotypes. Mild patients are usually asymptomatic; severe cases may suffer life-threatening hemolytic crises and even death (3). The classic triad of clinical symptoms consists of anemia, jaundice and splenomegaly (4). Epidemiologically, HS prevalence differs drastically across ethnic groups. Its incidence is comparatively high among Northern European and North American populations (1/5,000 to 1/2,000) (5), while a 2015 study by Wang et al. (6) calculated lower prevalence rates in Chinese individuals: 1.27 per 100,000 for males and 1.49 per 100,000 for females. Reliable epidemiological data focusing specifically on Chinese children are still insufficient to date.

At present, the eosin-5′-maleimide (EMA) flow cytometric assay serves as the mainstream preliminary screening test for pediatric HS. Featuring high sensitivity and specificity, the EMA test is routinely applied as a frontline screening tool prior to genetic verification (7). However, since this assay is not routinely available in most hospitals across China, this test was not performed in the present study.

Advancements in molecular diagnostic techniques have rendered genetic testing indispensable for the definitive diagnosis of hereditary disorders, especially for infant and neonatal patients (8). Next-generation sequencing (NGS) enables comprehensive screening of all genes linked to erythrocyte membrane disorders and precise localization of pathogenic variant sites, which facilitates definitive genetic confirmation of HS (9, 10).

Global HS mutational spectra exhibit obvious disparities across populations. *ANK1* and *SPTB* mutations are globally the most prevalent causative variants, followed by alterations in *SLC4A1* (2, 11). In contrast, variants in *SPTA1* and *EPB42* are considered rare; relevant published records are largely limited to Japan, South Korea and our previous single-center research (2, 12, 13). Nevertheless, differing from prior investigations, a relatively high prevalence of *SPTA1* pathogenic variants was observed in our study.

## 2 Materials and methods

This study was approved by the Ethics Committee of the First Affiliated Hospital of the Naval Military Medical University (CHEC2022-066). Before clinical evaluation and blood sample collection, written consent was obtained from the guardians of all children.

### 2.1 Subjects and clinical data collection

This was a retrospective study. A retrospective analysis was performed on 48 hospitalized children with HS admitted to the Department of Pediatrics, the First Affiliated Hospital of Naval Medical University, from May 2016 to March 2024. All the children were from unrelated families. Among them, 28 children with *de novo* mutations were reported in our previous study (13). We analyzed the clinical manifestations, physical examinations, family histories, and relevant laboratory test results of all children. All children underwent routine blood tests (including microscopic examination of red blood cell morphology and reticulocyte count), routine urine tests, blood biochemistry tests (including total, direct, and indirect bilirubin), ultrasonography of gallbladder, liver, and spleen, acidified glycerol lysis test, erythrocyte osmotic fragility test, glucose-6-phosphate dehydrogenase and pyruvate kinase enzyme assays, hemoglobin electrophoresis, and autoantibody tests. All children underwent genetic sequencing, and if a suspected pathogenic mutation was detected, Sanger sequencing was performed on their parents. The diagnostic criteria for HS were based on those in the literature (12). Diagnostic criteria for anemia: mild anemia is defined as hemoglobin (Hb) levels ranging from the lower limit of the normal reference range to above 90 g/L; moderate anemia corresponds to Hb: 60–90 g/L; severe anemia is defined as Hb: 30–59 g/L (14). The reference ranges for normal hemoglobin were as follows: >90 g/L for 1-3 months of age, >100 g/L for 4-6 months, >110 g/L for 6-59 months, >115 g/L for 5-11 years, and >120 g/L for 12-14 years (14). Height and weight percentiles were assessed according to the Chinese height-and-weight percentile reference for children and adolescents aged 0-18 years (15).

Among the 48 children in this study, 40 were followed up by telephone (with the most extended follow-up duration being 9 years after discharge), with the cutoff date set as June 1, 2025. The information was collected regarding the children's symptoms after discharge, whether they had received blood transfusions or splenectomy treatment, and the presence of complications such as gallstones.

### 2.2 Methods

#### 2.2.1 Second-generation sequencing

A total of 4 mL of peripheral venous blood was collected from each child and placed in an EDTA anticoagulant tube. Genomic DNA was extracted using a DNA Extraction Kit [TIANGEN Biotech (Beijing) Co., Ltd.] and the BloodGen Midi Kit (CWBIO, China). A DNA library was constructed using the GenCap Custom Kit (MyGenostics, Beijing), and 271 congenital anemia-related genes (including five erythrocyte membrane protein-encoding genes associated with HS pathogenesis) were screened using gene capture technology. Biotin-labeled capture probes (80–120-mer) were used to cover all the exons in the non-repetitive regions. The average gene coverage of the target region was 94.86%, the average sequencing depth was 436.75×, 90.14% of the target region had a coverage >30×, and 60.29% of the target region had a coverage >200×. The enriched DNA library was sequenced using the Illumina HiSeq sequencing platform. The sequencing results were compared with the single-nucleotide polymorphism (SNP) database (http://www.ncbi.nlm.nih.gov/projects/SNP/), the 1000 Genomes Project (https://www.1000genomes.org), and the Exome Aggregation Consortium (ExAC) database (http://exac.broadinstitute.org/).

#### 2.2.2 Sanger sequencing

For HS-related gene mutations detected by second-generation sequencing, loci with a frequency ˂0.01 in the 1000 Genomes Project and ExAC databases that were predicted to be functionally deleterious were confirmed by Sanger sequencing. Genomic DNA was obtained from the fathers and mothers of the children for Sanger sequencing validation. Specific PCR amplification and PCR product purification were performed on the sample DNA, and the products were sequenced using the terminator cycle sequencing method on an ABI 3730XL sequencer. DNA sequences were compared with the corresponding GenBank (https://www.ncbi.nlm.nih.gov) reference sequences to identify mutation sites. The gene reference sequence transcripts were NM_001142446 (ANK1), NM_001024858 (SPTB), NM_003126 (SPTA1), NM_000342 (SLC4A1), and NM_000119 (EPB42), respectively.

#### 2.2.3 Pathogenicity Prediction of Mutation Sites and Protein Function Analysis

Protein Variation Effect Analyzer (PROVEAN, http://provean.jcvi.org/index.php), Sorting Intolerant From Tolerant (SIFT, http://sift.jcvi.org), PolyPhen-2 (http://genetics.bwh.harvard.edu/pph2), and MutationTaster (http://mutationtaster.org) were used for the predictive analysis of protein and structural hazards. To predict the effect of genes on protein structure, we used the following three-dimensional protein structure modeling resource (http://www.proteinmodelportal.org).

### 2.3 Statistical analysis

Statistical analyses were performed using the Statistical Package for the Social Sciences software (version 23.0). Measurement data with non-normal distribution are expressed as median (25th and 75th percentile) [M (P25, P75)], and non-parametric tests were used for inter-group comparisons. Count data are expressed as percentages (%), and chi-square tests (*χ*^2^) were used for inter-group comparisons. Comparisons among multiple groups were performed using the Kruskal–Wallis test, and a *P*-value <0.05 was considered statistically significant.

## 3 Results

### 3.1 Clinical features and laboratory examination results of children with HS

The clinical manifestations and laboratory examination results of the 48 pediatric patients with HS are presented in Table 1.

**Table 1:** Clinical manifestations and laboratory examination results of 48 children with HS.

Group	Total (n)	Gender Male/Female	Age at diagnosis (months)	HB (g/L)	MCV (fL)	MCHC (g/L)	RET (%)	Total Bilirubin(*μ*mol/L)	History of Neonatal Jaundice (*n*, %)	History of Blood Transfusion (*n*, %)	Splenomegaly (*n*, %)
Mild Anemia	14	6/8	93.5 (26, 110.75)	96.5 (93.5, 102.25)	80.2 (73.65, 84.7)	351.5 (324.5, 359.25)	8.32 (1.83, 13.47)	58.5 (9.125, 64.75)	7 (50.0%)	1 (7.1%)	7 (50.0%)
Moderate Anemia	21	7/14	27 (4, 79.50)	71 (62, 77.5)	86.5 (78.5, 91.35)	330 (324, 342)	10.46 (6.545, 13.095)	38.8 (26.8, 71.7)	13 (61.9%)	15 (71.4%)	12 (57.1%)
Severe Anemia	13	7/6	37 (3, 70.5)	51 (42.5, 56.5)	84.9 (83.8, 91.35)	319 (308, 331)	10.3 (7.185, 16.74)	60.1 (31.45, 89.2)	10 (76.9%)	12 (92.3%)	7 (53.9%)
Test Value			*H* = 7.095	*H* = 41.00	*H* = 5.206	*H* = 9.055	*H* = 2.377	*H* = 2.284	*X*^2^ = 2.090	*X*^2^ = 22.749	*X*^2^ = 0.173
DF			2	2	2	2	2	2	2		
*P*-value			0.029	0.00	0.074	0.011	0.305	0.319	0.352	0.00	0.917

HB, mean Hemoglobin; MCHC, mean corpuscular hemoglobin concentration; MCV, mean corpuscular volume; RET, mean Reticulocyte.

Among all patients, there were 20 males (41.7%) and 28 females (58.3%), with a non-significant gender difference. All patients were ˂14 years at consultation, with the maximum age of 162 months. Among them, 38 cases (38/48, 79.2%) developed symptoms within 1 year of age; however, the median age at definitive diagnosis of HS was 48 months. For the 48 patients with HS, median hemoglobin concentration was 71.5 g/L, median mean corpuscular volume was 84.5 fL, median mean corpuscular hemoglobin concentration (MCHC) was 330 g/L, median reticulocyte count was 10.045%, and the median total bilirubin level was 45.2 µmol/L. A total of 30 cases (30/48, 62.5%) had a history of neonatal jaundice, which was unrelated to subsequent anemia severity. In total, 28 cases (28/48, 58.3%) had a blood transfusion history, and 26 (26/48, 54.2%) had splenomegaly. Following classification based on the minimum hemoglobin level of the patients, 14 (14/48, 29.2%) were diagnosed with mild anemia, 21 (21/48, 43.7%) with moderate anemia, and 13 (13/48, 27.1%) with severe anemia. The age at diagnosis of moderate-to-severe anemia was younger than that of mild anemia. More patients with moderate-to-severe anemia had a history of blood transfusion with lower MCHC values than those with mild anemia, with statistically significant differences.

### 3.2 Gene mutation characteristics of pediatric patients with HS in China

The gene mutation characteristics of the 48 Chinese pediatric patients with HS are illustrated in Table 2. The human reference assembly GRCh37/hg19 was adopted for identification of genomic variants. All identified variants were assessed and categorized as pathogenic or likely pathogenic following the ACMG standard variant pathogenicity classification guidelines.

**Table 2:** Gene mutation characteristics of 48 Chinese children with HS.

Patients	Gender	Age (months)	Gene	Exon/ intron	cDNA	Protein	Variant type	Variation source
	M	89	SPTB	Exon7	c.876_880del	p.Lys292Asnfs*2	Frameshift	Mother
	F	48	SPTB	Exon18	c.3866G>A	p.Trp1289 Ter	Nonsense	Denovo
	F	27	SPTB	Exon22	c.4885C>A	p.Gln1629Ter	Nonsense/	Mother
	M	96	SPTB	Exon15	c.3103C>T	p.Arg1035Trp	Missense	Mother
	M	32	SPTB	Exon 15	c.3471G>A	p.Trp1157Ter	Nonsense	Mother
	M	15	SPTB	Exon3	c.376C>T	p.Gln126Ter	Nonsense	Denovo
	F	2	SPTB	Exon26	c.5530G>T	p.Glu1844Ter	Nonsense	Father
	F	113	SPTB	Exon 19	c.4105A>G	p.Lys1369Glu	Missense	Mother
	M	5	SPTB	Exon 11	c.1249C>T	p.Gln417 Ter	Nonsense	Father
	M	8	SPTB	Exon29	c.5970G>C	p.Arg1900Ser	Missense	Father
1	F	5	SPTB	Exon15	c.2856_2862delAG CCCTC	p.Ala953Glufs*7	Frameshift	Father
2	M	95	SPTB	Exon2	c.148+2T>C	—	Splicing error	Denovo
3	F	94	SPTB	Exon5	c.475_523delTG CTACCCTC AGATTCAG GACATTGT GGTCCAAA CTCAGGAA GGTCGTGA AACACGCTCAGAGGACATTGTG GTCCAAAC TCAG	p.Ile159fs	Frameshift	Denovo
4	F	92	SPTB	Exon14	c.2494C>T	p.Gln832Ter	Nonsense	Denovo
5	M	57	SPTB	Exon16	c.3520C>T	p. Gln1174Ter	Nonsense	Denovo
6	F	113	SPTB	intron11	c.1342-10G>A	—	Splicing error	Denovo
7	F	39	SPTB	Exon16	c.3450G>A	p.Trp1150Ter	Nonsense	Denovo
8	M	7	SPTB	Exon8	c.866G>A	p.Arg289His	Missense	Mother
9	M	162	SPTB	Exon23	c.4770C>G	p.Tyr1590Ter	Nonsense	Denovo
10	F	7	SPTB	Exon3	c.218C>T	p.Arg70Ter	Nonsense	Denovo
11	F	49	SPTB	Exon23	c.4581del	p.Leu1528Trpfs	Frameshift	Mother
	M	63	ANK 1	Exon4	c.399T>G	p.Tyr133Ter	Nonsense/ PTC	*De novo*
	F	2	ANK 1	Exon17	c.1914_1918delTTTGC	p.Pro638Profs*14	Frameshift/PTC	Mother
	M	8	ANK 1	Exon 14	c.1564delC	p.Leu522Cysfs*44	Frameshift/PTC	Denovo
	F	3	ANK 1	Exon37	c.4439dupA	p.Asn1480Lys20	Frameshift/PTC	Father
	M	110	ANK 1	Exon37	c.4510_4513del	p.Asn1504Trpfs*17	Frameshift/PTC	Father
	F	95	ANK 1	Exon27	c.2961delC	p.Thr988Profs*30	Frameshift/PTC	Father
	F	107	ANK 1	Exon18	c.2142dupT	p.Pro715Serfs*111	Frameshift/PTC	Mother
	M	103	ANK 1	Intron26	c.2858+1G>C	—	Splicing error	Father
	F	4	ANK 1	Exon28	c.3235delG	p.Glu1079fs	Frameshift/PTC	Denovo
	M	49	ANK 1	Exon39	c.4739A>G	p.Gln1580Arg	Missense/ AA	Mother
	F	84	ANK 1	Exon25	c.2638-2A>G	—	Splicing error	Denovo
	M	3	ANK 1	Exon27	c. 2926C>T	p.Arg976Ter	Nonsense/ PTC	Denovo
	M	37	ANK 1	Exon34	c.4153C>T	p.Arg1385Ter	Nonsense/ PTC	Mother
12	F	48	ANK 1	Exon14	c.1504-9G>A	—	Splicing error	Father
13	F	114	ANK 1	Exon25	c.2638_2639delGCinsTA	p.Ala880Ter	Nonsense	Denovo
14	F	70	ANK 1	Exon 32	c.3925C>T	p.Gln1309Ter	Nonsense	Denovo
15	F	65	ANK 1	EExon 3	c.202G>T	p.Glu68Ter	Nonsense	Mother
16	M	3	ANK 1	Exon 28	c.3235G>T	p.Glu1079Ter	Nonsense	Mother
	M	122	SLC4 A1	Exon18	c.2423G>A	p.Arg808His	Missense	Denovo
	F	13	SLC4 A1	Exon12	c.1388G>A	p.Gly463Asp	Missense	Mother
	F	83	SLC4 A1	Exon16	c.1979C>T	p.Pro660Leu	Missense	Denovo
17	M	2	SLC4 A1	Exon12	c.1414G>C	p.Glu472Gln	Missense	Mother
18	F	3	SLC4 A1	Exon 18	c.2423G>A	p.Arg808His	Missense	Father
	F	3	SPTA 1	Exon33	c.4823G>A	p.Arg1608His	Missense/AA	Mother
	F	4	SPTA 1	Exon30	c.4418C>T	p.Thr1473Met	Missense/AA	Father
19	F	105	SPTA 1	Exon14	c.1795A>T	p.Asn599Tyr	Missense	Mother
20	F	4	SPTA 1	Exon2	c.83G>A	p.Arg28His	Missense	Denovo

In the table, patients marked with a preceding number are those with denovo mutations identified in this study, while those without a number are cases previously reported in our research. F denotes female, M denotes male.

Among 48 patients, 20 were males (41.7%), and 28 were females (58.3%). Besides the 28 novel mutations reported previously (9), 13 of the remaining 20 cases were novel mutations, and 7 were known mutation loci that had been previously reported.

A total of 28 cases (28/48, 58.3%) had a family history of HS, of which 17 (17/28, 60.7%) were maternally inherited, and 11 (11/28, 39.3%) were paternally inherited. Among all mutation types, there were 18 cases of nonsense (18/48, 37.5%), 14 cases of missense (14/48, 29.2%), 11 cases of frameshift (11/48, 22.9%), and 5 cases of splicing (5/48, 10.4%) mutations.

### 3.3 Comparison of clinical features and laboratory examination results in pediatric patients with different coding gene mutations

A comparison of the clinical symptoms in pediatric patients with different coding gene mutations is presented in Table 3.

**Table 3:** Clinical characteristics and laboratory examination results of children with different types of gene mutations.

Mutated gene	Total (n)	Gender F/M	Age (months)	HB (g/L)	MCV(fL)	MCHC (g/L)	RET (%)	TBil μmol/L	History of Neonatal Jaundice (*n*, %)	History of Blood Transfusion (*n*, %)	Splenomegaly (*n*, %)
SPTB	21	10/11	48 (7.5, 94.5)	84 (62, 98)	79.9 (78.5, 85.1)	341 (321.5, 354.5)	9.87 (5.435, 13.425)	42.3 (20.45, 68.35)	11 (52.4%)	8 (38.1%)	12 (57.1%)
ANK 1	18	8/10	56 (3.75, 97)	61.5 (52.5, 79.25)	85.7 (83.25, 89.9)	329.5 (310.75，345)	13.31 (10.13, 17.42)	59.5 (38.28, 100)	13 (72.2%)	16 (88.9%)	12 (66.7%)
SLC4 A1	5	2/3	13 (2.5, 102.5)	67 (62.5, 81.5)	89.4 (80.15, 101.5)	347 (294, 361)	6.09 (4.84, 7.7)	34.2 (20.4, 60.65)	3 (60.0%)	3 (60.0%)	2 (40.0%)
SPTA 1	4	0/4	4 (3.25, 79.75)	66.5 (50.75, 90.5)	80.35 (65.65, 92.8)	314 (297.25, 348.75)	3.23 (1.12, 7.99)	32.15 (13.75, 70.95)	3 (75.0%)	1 (25.0%)	0 (0.0%)
Test Value			*H* = 1.930	*H* = 5.698	*H* = 6.457	*H* = 2.778	*H* = 16.256	*H* = 5.710	*X*^2^ = 1.923	*X*^2^ = 12.287	*X*^2^ = 6.339
DF			3	3	3	3	3	3	3	3	3
*P*-value			0.587	0.127	0.091	0.427	0.001	0.127	0.588	0.006	0.096

F, mean female; M, mean male; HB, mean Hemoglobin; MCHC, mean corpuscular hemoglobin concentration; MCV, mean corpuscular volume; RET, mean Reticulocyte; TiBL, mean Total Bilirubin.

Among 48 Chinese children with HS gene mutations, SPTB gene mutations were the most frequent, accounting for 21 cases (21/48, 43.8%), followed by *ANK1* gene mutations in 18 cases (18/48, 37.5%), *SLC4A1* gene mutations in 5 cases (5/48, 10.4%), and *SPTA1* gene mutations in 4 cases (4/48, 8.3%). The median hemoglobin concentration was lowest, and the median total bilirubin level was highest in patients with ANK1 mutations; however, statistically non-significant differences were observed. Conversely, the reticulocyte percentage and proportion of patients with a blood transfusion history were significantly higher in the ANK1 mutation group (*P* < 0.05).

### 3.4 Comparison of clinical symptoms in pediatric patients with different mutation types

A comparison of the clinical symptoms in pediatric patients with different types of mutation is presented in Table 4.

**Table 4:** Clinical manifestations and laboratory examination results of children with different mutation types.

Mutation type	Total (n)	Gender F/M	Age (months)	HB (g/L)	MCV (fL)	MCHC (g/L)	RET (%)	TBil μmol/L	History of Neonatal Jaundice (*n*, %)	History of Blood Transfusion (*n*, %)	Splenomegaly (*n*, %)
Nonsense mutation	18	9/9	38 (6.5, 66.25)	68 (54.75, 95.25)	84.7 (79.5, 88.98)	331 (322.25, 350.25)	11.97 (7.94, 15.83)	61.15 (28.53, 93.8)	14 (77.8%)	13 (72.2%)	11 (61.1%)
Missense mutation	14	6/8	10.5 (3.75, 98.25)	70 (56.5, 93)	82.25 (69.83, 93.2)	324 (294, 350.5)	5.12 (1.44, 9.1)	30.2 (9.13, 55.2)	7 (50.0%)	6 (42.9%)	4 (28.6%)
Frameshift mutation	11	3/8	49 (4, 95)	72 (60, 87)	84 (78.3, 94.9)	341 (333, 359)	11.81 (9.87, 15.52)	48 (36.7, 63.4)	5 (45.5%)	6 (54.5%)	6 (54.5%)
Splicing mutation	5	2/3	95 (66, 108)	71 (59, 99)	87.5 (82.5, 91.55)	324 (306.5, 356)	16.76 (10.43, 22.15)	63 (25.7, 76.95)	4 (80.0%)	3 (60.0%)	5 (100%)
Test Value			*H* = 3.813	*H* = 0.329	*H* = 2.825	*H* = 4.649	*H* = 17.227	*H* = 5.354	*X*^2^ = 4.743	*X*^2^ = 2.879	*X*^2^ = 8.275
DF			3	3	3	3	3	3	3	3	3
*P*-value			0.282	0.955	0.419	0.199	0.001	0.148	0.192	0.411	0.041

F, mean female; M, mean male; HB, mean Hemoglobin; MCHC, mean corpuscular hemoglobin concentration; MCV, mean corpuscular volume; RET, mean Reticulocyte; TiBL, mean Total Bilirubin.

Among the 48 Chinese children with HS gene mutations, nonsense mutations were the most common, accounting for 18 cases (18/48, 37.5%), followed by missense mutations in 14 cases (14/48, 29.2%), frameshift mutations in 11 cases (11/48, 22.9%), and splicing mutations in 5 cases (5/48, 10.4%). The splicing mutation group had the highest reticulocyte percentage and the highest splenomegaly proportion. Conversely, the missense mutation group had the lowest reticulocyte percentage and lowest splenomegaly proportion, with statistically significant differences (*P* < 0.05).

### 3.5 Distribution characteristics of mutation loci in the anchor region of SPTB, ANK1, SPTA1, and SLC41 in 48 pediatric patients with HS

The SPTB mutation loci distribution in 21 Chinese children is presented in Figure 1.

**Figure 1:**
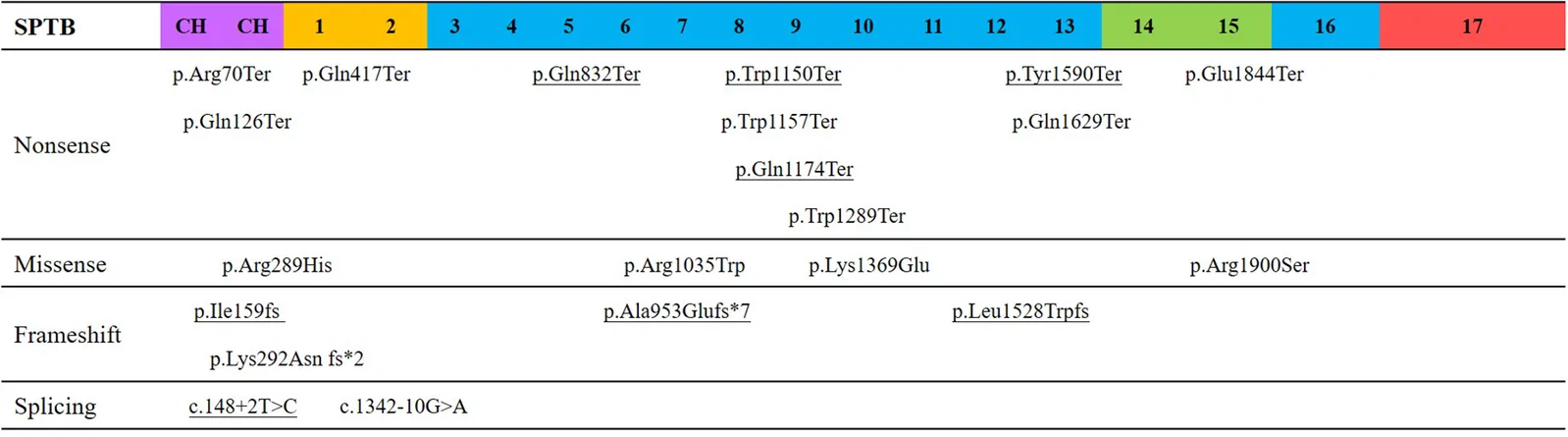
Schematic diagram of the SPTB anchoring domain Abbreviations: In the figure, the purple region represents the actin-binding domain, the orange region the dimerization domain, the blue region a segment of the spectrin repeat domain, the green region the ankyrin-binding domain, and the red region the tetramerization domain. Underlined sites indicate newly identified mutation loci that have not been previously reported.

Six mutations were in the actin-binding domain (3 cases of moderate anemia, including 1 nonsense and 2 frameshift mutations; 3 cases of mild anemia, including 1 nonsense, 1 missense, and 1 splicing mutation), among which 2 were novel mutations. Two mutations were in the dimerization domain (1 case of severe anemia with a nonsense mutation; 1 case of mild anemia with a splicing mutation). Twelve mutations were located in the spectrin repeat domain (3 cases of severe anemia, including 2 nonsense and 1 missense mutation; 4 cases of moderate anemia, including 2 nonsense and 2 frameshift mutations; 5 cases of mild anemia, including 3 nonsense and 2 missense mutations), among which 6 were novel mutations. One mutation was in the ankyrin-binding domain and presented as moderate anemia with a nonsense mutation. Among these 21 mutations, 11 were nonsense mutations (3 cases of severe, 4 cases of moderate, and 4 cases of mild anemia), 4 were missense mutations (1 case of severe and 3 cases of moderate anemia), 4 were frameshift mutations (all were moderate anemia), and 2 were splicing mutations (all were mild anemia).

The ANK1 mutation loci distribution in the anchor region of the 18 Chinese children is presented in Figure 2 Among them, 6 mutations were in the N-terminal region (1 case of severe anemia with a frameshift mutation; 4 cases of moderate anemia, including 2 frameshift, 1 nonsense, and 1 splicing mutation; 1 case of mild anemia with a nonsense mutation), with 2 being novel mutations. Seven mutations were in the central domain (3 cases of severe anemia, including 2 nonsense and 1 splicing mutation; 3 cases of moderate anemia, including 1 frameshift, 1 nonsense, and 1 splicing mutation; 1 case of mild anemia with a frameshift mutation), with 1 being a novel mutation. Five mutations were in the C-terminal regulatory domain (3 cases of severe anemia, including 1 frameshift, 1 nonsense, and 1 missense mutation; 1 case of moderate anemia with a nonsense mutation; 1 case of mild anemia with a frameshift mutation). Among these 18 mutations, 7 were frameshift mutations (2 cases of severe, 3 cases of moderate, and 2 cases of mild anemia), 7 were nonsense mutations (3 cases of severe, 3 cases of moderate, and 1 case of mild anemia), 3 were splicing mutations (1 case of severe and 2 cases of moderate anemia), and 1 was a missense mutation (1 case of severe anemia).

**Figure 2:**
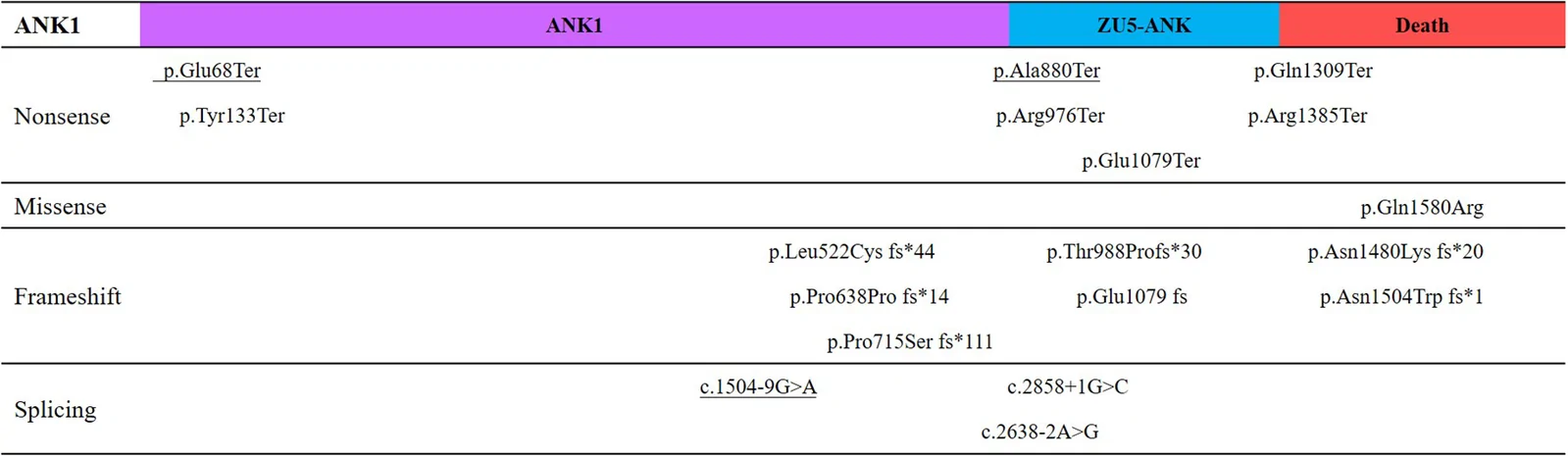
Schematic diagram of the ANK1 anchoring domain Abbreviations: In the figure, the green region denotes the N-terminal domain, the blue region the central domain, and the red region the C-terminal regulatory region. Underlined sites indicate newly identified mutation loci that have not been previously reported.

Figures 3, 4 exhibit gene mutation loci distribution in all children with mutations in SLC4A1 and SPTA1.The mutation loci of the five children with SLC4A1 mutations were located in the HCO_3_ region, all of which were missense mutations (4 cases of moderate and 1 case of mild anemia), with 1 case identified as a novel mutation. The mutation loci of the 4 children with SPTA1 mutations were all located in the spectrin region, all were missense mutations (2 cases of severe, 1 case of moderate, and 1 case of mild anemia), among which 1 case was a novel mutation.

**Figure 3:**
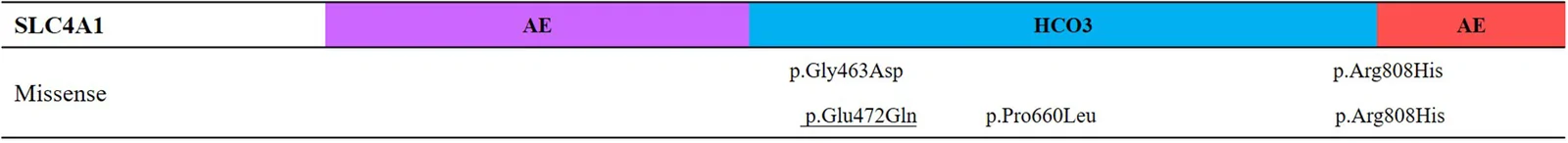
Schematic diagram of the SLC4A1 anchoring domain Abbreviations: In the figure, the yellow region denotes the anion exchange protein domain, and the red region the HCO3⁻ transporter domain. Underlined sites indicate newly identified mutation loci that have not been previously reported.

**Figure 4:**
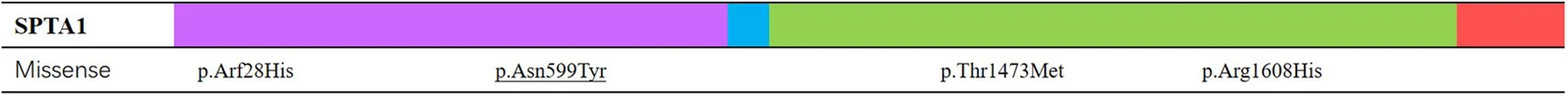
Schematic diagram of the SPTA1 anchoring domain Abbreviations: In the figure, the red region denotes the spectrin repeat domain, the orange region the Src homology 3 domain, the purple region the EF-hand domain, and the yellow region the Ca^2^⁺-insensitive EF-hand domain. Underlined sites indicate newly identified mutation loci that have not been previously reported.

Given that most “domain-disease severity” subgroups only contained 1-3 cases, we cannot draw definitive conclusions regarding the definite association between each domain and disease severity.

### 3.6 Onset of disease, treatment, and long-term growth and development Status of 40 Chinese pediatric patients with HS after discharge

Telephone follow-up was conducted for 48 pediatric patients, among whom 8 cases were lost to contact, and 40 cases (40/48, 83.3%) were successfully followed up. The follow-up duration ranged from 15 to 109 months after discharge, with a mean of 68.1 months. By the end of the follow-up period, 12 cases (12/40, 30.0%) had undergone splenectomy, and 2 cases (2/40, 5.0%) were scheduled to undergo splenectomy in 6 months, accounting for 35% of all successfully followed-up patients.

The Analysis of clinical profiles in children after splenectomy is presented in Table 5. Among 12 patients who had undergone splenectomy, 9 were diagnosed with severe anemia at initial presentation and 3 with moderate anemia. Of these 12 cases, none of the children had height below the age- and sex-specific P3. After splenectomy, all patients revealed a significant increase in hemoglobin levels than preoperative values (*P* < 0.05), with no need for blood transfusion postoperatively, a marked improvement in exercise tolerance, and no gallstone formation.

**Table 5:** Analysis of clinical profiles in children after splenectomy.

Anemia severity	Total (*n*, %)	Age at surgery (years, median)	Number of cases with normal Hb after surgery (*n*, %)	Postoperative blood transfusion (n)	Gallstones (n)	Height < P50 (*n*, %)	Height < P3 (*n*, %)	Number of cases with improved exercise tolerance after surgery (*n*, %)
Moderate anemia	3 (25%)	9 (9)	3 (100%)	0	0	3 (100%)	0 (0%)	3 (100%)
Severe anemia	9 (75%)	7 (6.3)	9 (100%)	0	0	7 (77.8%)	0 (0%)	9 (100%)

The Analysis of clinical profiles in non-splenectomized children is presented in Table 6. At initial diagnosis, 6 cases (6/26, 23.0%) were identified with severe, 10 cases (10/26, 38.5%) with moderate, and 10 cases (10/26, 38.5%) with mild anemia. At follow-up, one case of severe anemia had gallstones (currently 8 years and 1 month old, with a post-discharge period of 28 months). Twenty cases (20/26, 76.9%) underwent regular blood routine tests, whereas six cases (6/26, 23.1%) did not undergo any relevant examinations after discharge. Only 6 cases (6/26, 23.1%) underwent regular hepatosplenic ultrasonography, all of whom had splenomegaly. Among the 26 non-splenectomized children, 17 (17/26, 65.4%) had height below the age- and sex-specific P50. Compared with the splenectomized group (2/12), *χ*^2^ = 0.561, *P* = 0.455, showing no statistically significant difference. None of the patients in this subgroup had height below P3, suggesting that this disease exerted no obvious adverse effect on height.

**Table 6:** Analysis of clinical profiles in non-splenectomized children.

Anemia severity	Total (*n*, %)	Follow-up time (months, mean)	Number of cases with normal Hb (n)	Blood transfusion (n)	Gallstones (n)	Height > P50 (*n*, %)	Height < P3 (*n*, %)	Impact of exercise tolerance after discharge (*n*, %)
Mild anemia	10 (38.5%)	68.8	7 (7/7)	0	0	4 (40.0%)	0 (0%)	0 (0%)
Moderate anemia	10 (38.5%)	63.4	2 (2/8)	2	0	3 (30.0%)	0 (0%)	5 (50.0%)
Severe anemia	6 (23%)	88.2	0 (0/5)	3	1	2 (33.3%)	0 (0%)	6 (100%)

Some patients were lost to this specific follow-up data point for hemoglobin re-measurement; thus, denominators for “Number of cases with normal Hb” are smaller than the total number of patients in each group.

Among the 40 followed-up patients, 7 families had another child subsequently, of whom 2 were diagnosed with hemolytic anemia upon examination; both children carried familial gene mutation loci. The guardians of all patients reported that the definitive diagnosis made at our hospital alleviated their economic and psychological burdens to a certain extent.

## 4 Discussion

HS is a hereditary hemolytic anemia caused by abnormalities in erythrocyte membrane proteins. Its clinical manifestations demonstrate marked heterogeneity, ranging from mild to severe (16). Significant differences in clinical phenotypes may occur among individuals with the same allelic mutation. Consequently, HS is prone to missed diagnosis and misdiagnosis in adults and children (17, 18). In this study, all pediatric patients with anemia (48/48, 100%), 30 cases (30/48, 62.5%) had jaundice, and 26 cases (26/48, 54.2%) had splenomegaly, consistent with the main clinical manifestations reported in previous study (4).

No gallstones were detected in any patient at the initial diagnosis. This may be attributed to the fact that gallstone formation is a long-term chronic process. Simultaneously, the enrolled patients were relatively young at consultation, which was insufficient for gallstone development. During the follow-up of non-splenectomized patients, one case of severe anemia (currently 8 years and 1 month old) developed gallstones, suggesting that gallstone risk increases with age in untreated patients with HS.

A Recent study have demonstrated that HS with infant-onset is frequently associated with more severe clinical phenotypes in children under 14 years of age (19). Consistent with these reports, our cohort showed that children with moderate-to-severe anemia were diagnosed at an earlier age and carried a higher transfusion burden compared with patients with mild anemia. Lower MCHC, a hallmark of red-cell membrane defects in HS, was also prominent among patients with moderate-to-severe anemia. This finding is biologically plausible: severe membrane damage accelerates extravascular hemolysis, which manifests as more profound anemia, earlier clinical recognition, and greater transfusion requirements. In longitudinal follow-up, patients presenting at younger ages were more likely to require repeated transfusions, and several developed transfusion-dependence prior to splenectomy. Of note, non-splenectomized children exhibited evident growth lag relative to age-matched peers. Early-onset chronic hemolytic anemia may exert irreversible adverse effects on physical growth, which cannot be fully rescued by later supportive interventions. Therefore, long-term prospective surveillance is critical to characterize growth-related outcomes in pediatric HS.

HS is caused by deficiency or dysfunction of erythrocyte membrane cytoskeletal proteins (ankyrin, *α*-spectrin, *β*-spectrin, protein 4.2, and band 3 protein), which are encoded by the *ANK1*, *SPTA1*, *SPTB*, *EPB42*, and *SLC4A1* genes, respectively (20). In previous study, with relatively large sample sizes, ANK1 mutations were the most common, followed by SPTB and SLC4A1 mutations, whereas SPTA1 and EPB42 mutations were relatively rare (6). Among the 48 Chinese pediatric patients with HS included in our study, SPTB mutations were the most frequent (21/48, 43.8%), followed by ANK1 (18/48, 37.5%), SLC4A1 (5/48, 10.4%), and SPTA1 (4/48, 8.3%) mutations; however, no EPB42 mutations were detected. The high prevalence of SPTB mutations, together with four cases of SPTA1 mutations that were reported as rare in previous studies, suggests that this may represent a unique profile of gene mutation distribution in Chinese pediatric patients with HS. However, this finding may also be attributed to the relatively small sample size of this study, which therefore warrants validation in a larger cohort of Chinese pediatric patients with HS in future studies. A previous study has indicated that ANK1 mutations-caused HS is associated with more severe anemia than that caused by SPTB mutations (21). Our findings suggest an association between mutated genes and clinical manifestations. The SPTB mutation group had a higher median hemoglobin level than the other three gene mutation groups. Conversely, patients with ANK1 mutations had the lowest median hemoglobin concentration and the highest total bilirubin level, although these differences were statistically non-significant. Notably, the reticulocyte proportion and blood transfusion history rate were significantly higher in ANK1 mutation group, suggesting that pediatric patients with HS and ANK1 mutations tend to present with more severe clinical symptoms.

Among HS mutation types, nonsense, missense, frameshift, and splicing mutations are predominant (22, 23), while other mutation types are relatively rare. In this study, nonsense mutations were the most frequent (18/48, 37.5%), followed by missense (14/48, 29.2%), frameshift (11/48, 22.9%), and splicing (5/48, 10.4%) mutations, consistent with previous reports. A comparison of clinical manifestations across mutation types revealed that the splicing mutation group had the highest reticulocyte percentage and the highest splenomegaly proportion. However, the missense mutation group had the lowest reticulocyte percentage and the lowest splenomegaly proportion, with statistically significant differences in both comparisons. These findings suggest that splicing mutations induce more significant alterations in gene function, which may lead to more severe clinical symptoms, whereas missense mutations exert the opposite effect. The above difference is likely related to the fact that missense mutations tend to maintain partial physiological functions of the protein, while nonsense mutations, frameshift mutations and splicing aberrations mostly cause protein truncation or deficient protein expression. However, due to the limited sample size of each subgroup in this study, all stratified comparative analyses are exploratory only, and the corresponding conclusions may not be widely generalized to broader populations.

In the past, most hospitals relied on clinical manifestations and routine laboratory tests to diagnose HS (7). Owing to conventional detection method limitations for diagnosing this disease and the relatively limited understanding of pediatric HS among some clinicians, it is challenging to make a timely diagnosis at the initial onset of symptoms (21). Some pediatric patients with HS were misdiagnosed for a long time and received inappropriate treatment. In our study, most patients (38/48, 79.2%) developed symptoms at 1 year of age; however, the median age at the definitive diagnosis was 48 months. The diagnosis of some patients took 3 years or longer. Delayed diagnosis adversely affects the treatment and prognosis of patients and imposes considerable economic and psychological burdens on their parents. Our follow-up data confirmed this. We observed that the earlier the diagnosis was made, the earlier the subsequent treatment (blood transfusion or splenectomy) was administered by parents; such patients revealed almost no difference in growth and development compared with age-matched peers, and the incidence of complications (gallstones) was also reduced. Consequently, an early definitive diagnosis is of great significance for pediatric patients with HS treatment and prognosis.

With advances in medical technology and widespread clinical application of molecular genetic testing, NGS has emerged as a powerful tool for the clinical diagnosis of pediatric HS (8, 9). Among all patients with HS included in this study, most had undergone multiple consultations at other hospitals without a confirmed etiology. Through a combination of routine laboratory and genetic testing, we identified HS-related pathogenic gene mutations in all patients, some of whom had a positive family history of HS. Based on the clinical manifestations and genetic testing results of patients, we concluded that these gene mutations were the pathogenic factors underlying the disease in all the enrolled patients, thereby clarifying the etiology and confirming the diagnosis. Accordingly, we propose that NGS should be more extensively applied in the definitive diagnosis of pediatric HS, as it holds great significance for the early diagnosis of HS in children.

The treatment for pediatric HS mainly consists of blood transfusion and splenectomy (24). However, during long-term and repeated blood transfusions, excessive iron accumulation may pose a greater potential threat than anemia, which could lead to multiple organ failure and death (18). Consequently, splenectomy has become the first-line treatment option for most pediatric patients with HS (12). Nevertheless, some patients exhibit non-significant improvement in anemia symptoms after splenectomy and still require long-term regular blood transfusion therapy (25). For these patients, gene therapy may become the primary or even the only curative treatment modality in the future. Our study enriches the mutational spectrum of hereditary spherocytosis among Chinese pediatric patients and offers theoretical support for the future development of gene-based therapies for childhood HS.

A total of 40 pediatric patients were successfully followed up in this study, with the longest post-discharge follow-up duration reaching nine years, which has rarely been reported in existing studies on pediatric HS. We identified that among the 12 patients who subsequently underwent splenectomy, the hemoglobin levels re-examined after surgery increased significantly compared with preoperative values. None of them required further blood transfusion therapy. None of the children who underwent splenectomy in this study developed complications such as gallstones or reduced exercise tolerance their height was comparable to those of age-matched peers. However, multiple confounding factors may affect physical development, so we cannot draw a causal conclusion that splenectomy improves growth and development. Conversely, among the non-splenectomized patients, Seventeen patients (65.4%) had heights below the median height (P50) for children of the same age and sex, and one case of severe anemia was diagnosed with gallstones at 8 years and 1 month. Therefore, regardless of the severity of anemia in pediatric patients with HS, regular monitoring of growth and development levels and timely early intervention are of great importance. It is also unfortunate that 2 out of the 7 children born to the families of the enrolled patients were still diagnosed with hemolytic anemia, which could have been avoided through prenatal diagnostic techniques or assisted reproductive technologies. We acknowledge that there remain deficiencies in our post-discharge follow-up work. Although we provided guidance on subsequent treatment during the patients' hospitalization and one month after discharge, and also reminded parents to receive genetic counseling when planning another pregnancy, some parents had insufficient disease awareness and misunderstandings about the treatment plan, leading to inadequate standardization of re-examinations and treatment after discharge. In the future, we will implement more standardized follow-up and guidance in the post-discharge management of pediatric patients with HS to improve their long-term prognosis. All follow-up assessments in this study were completed through telephone interviews only. Unlike face-to-face outpatient reviews, this method may lead to recall bias and data inaccuracies when guardians report their children's clinical information. To reduce such potential biases, we exclusively contacted primary caregivers who live with the patients long-term and have full knowledge of their follow-up examination results. This selection criterion helped minimize errors arising from insufficient familiarity with the children's disease status. Even so, telephone follow-up still has inherent limitations compared with on-site clinical evaluations. In addition, indicators of exercise tolerance were solely reported subjectively by the children's guardians rather than objective quantitative clinical measurements.

We attempted to establish a prognostic risk stratification system for pediatric HS by integrating disease-causing genes, mutation types, and structural variant characteristics, to predict disease severity, transfusion dependence, and splenectomy requirement at the initial diagnosis. However, due to the limited sample size of the present study, subgroup classification based on combined gene and mutation typing resulted in zero cases in several stratified groups, which precluded valid statistical comparisons. We will further expand the sample size and conduct in-depth stratified analyses in future studies. This long-term exploration will help establish a more accurate prognostic model to support individualized risk prediction and clinical management for newly diagnosed pediatric HS patients.

In conclusion, as the largest Chinese single-center cohort with the longest reported follow-up of pediatric HS, this study assessed the clinical manifestations, laboratory test results, and genotypic characteristics of 48 Chinese pediatric patients with HS. This study provides a theoretical basis for expanding the pediatric HS gene database, providing genetic counseling to families of patients with HS planning subsequent pregnancies, and facilitating the gene therapy development for pediatric HS in the future. Meanwhile, this single-center study compensates for the deficiency of long-term follow-up in prior relevant clinical researches and offers clinical evidence for studies on the long-term prognosis of pediatric hemolytic anemia. For clinicians, regular post-discharge follow-up and guidance must also be highlighted, as this is of great significance for pediatric patients with HS and their families.

### 4.1 Limitations

This study has several limitations. First, all follow-up information was collected via telephone interviews, which may introduce recall bias from guardians' self-reported clinical data. Exercise tolerance was assessed subjectively by caregivers rather than objective quantitative measurements. Second, the overall sample size was relatively small. When patients were stratified by disease-causing gene combined with mutation type, several subgroups contained zero cases, making valid statistical comparisons infeasible. Therefore, we could not establish a prognostic risk-stratification model to predict disease severity, transfusion dependence and splenectomy requirement at initial diagnosis. Although our cohort represents one of the relatively long-term followed pediatric HS cohorts in China, multi-center, large-sample prospective studies are warranted to validate our results.

## Data Availability

The datasets presented in this article are not readily available due to patient privacy and ethical restrictions. An anonymized minimal dataset supporting the conclusions will be available from the corresponding author upon reasonable request, in compliance with institutional ethics approval.
